# Complete genome sequence of *Mahella australiensis* type strain (50-1 BON^T^)

**DOI:** 10.4056/sigs.1864526

**Published:** 2011-06-30

**Authors:** Johannes Sikorski, Hazuki Teshima, Matt Nolan, Susan Lucas, Nancy Hammon, Shweta Deshpande, Jan-Fang Cheng, Sam Pitluck, Konstantinos Liolios, Ioanna Pagani, Natalia Ivanova, Marcel Huntemann, Konstantinos Mavromatis, Galina Ovchinikova, Amrita Pati, Roxanne Tapia, Cliff Han, Lynne Goodwin, Amy Chen, Krishna Palaniappan, Miriam Land, Loren Hauser, Olivier D. Ngatchou-Djao, Manfred Rohde, Rüdiger Pukall, Stefan Spring, Birte Abt, Markus Göker, John C. Detter, Tanja Woyke, James Bristow, Victor Markowitz, Philip Hugenholtz, Jonathan A. Eisen, Nikos C. Kyrpides, Hans-Peter Klenk, Alla Lapidus

**Affiliations:** 1 DSMZ - German Collection of Microorganisms and Cell Cultures GmbH, Braunschweig, Germany; 2 DOE Joint Genome Institute, Walnut Creek, California, USA; 3 Los Alamos National Laboratory, Bioscience Division, Los Alamos, New Mexico, USA; 4 Biological Data Management and Technology Center, Lawrence Berkeley National Laboratory, Berkeley, California, USA; 5 Oak Ridge National Laboratory, Oak Ridge, Tennessee, USA; 6 HZI – Helmholtz Centre for Infection Research, Braunschweig, Germany; 7 Australian Centre for Ecogenomics, School of Chemistry and Molecular Biosciences, The University of Queensland, Brisbane, Australia; 8 University of California Davis Genome Center, Davis, California, USA

**Keywords:** strictly anaerobic, motile, spore-forming, Gram-positive, moderately thermophilic, chemoorganotrophic, *Thermoanaerobacteraceae*, GEBA

## Abstract

*Mahella australiensis* Bonilla Salinas *et al*. 2004 is the type species of the genus *Mahella*, which belongs to the family *Thermoanaerobacteraceae*. The species is of interest because it differs from other known anaerobic spore-forming bacteria in its G+C content, and in certain phenotypic traits, such as carbon source utilization and relationship to temperature. Moreover, it has been discussed that this species might be an indigenous member of petroleum and oil reservoirs. This is the first completed genome sequence of a member of the genus *Mahella* and the ninth completed type strain genome sequence from the family *Thermoanaerobacteraceae*. The 3,135,972 bp long genome with its 2,974 protein-coding and 59 RNA genes is a part of the *** G****enomic* *** E****ncyclopedia of* *** B****acteria and* *** A****rchaea * project.

## Introduction

Strain 50-1 BON^T^ (= DSM 15567 = CIP 107919) is the type strain of *Mahella australiensis*, and the type and only species of the monotypic genus *Mahella* [1,2]. The genus name is derived from the Neo-Latin word *Mahella* (named in honor of the American microbiologist R. A. Mah, for his important contribution to the taxonomy of anaerobes) [2]. The species epithet is derived from the Neo-Latin word *australiensis* (related to Australia) [1]. Strain 50-1 BON^T^ was isolated from the Riverslea Oil Field in the Bowen-Surat basin in Queensland, eastern Australia [1]. No further isolates have been reported for *M. australiensis*. Here we present a summary classification and a set of features for *M. australiensis* 50-1 BON^T^, together with the description of the complete genomic sequencing and annotation.

## Classification and features

A representative genomic 16S rRNA sequence of *M. australiensis* was compared using NCBI BLAST under default settings (e.g., considering only the high-scoring segment pairs (HSPs) from the best 250 hits) with the most recent release of the Greengenes database [3] and the relative frequencies, weighted by BLAST scores, of taxa and keywords (reduced to their stem [4] were determined. The three most frequent genera were *Clostridium* (76.6%), *Mahella* (18.5%) and *Pelotomaculum* (4.8%) (36 hits in total). Regarding the two hits to sequences from members of the species, the average identity within HSPs was 99.9%, whereas the average coverage by HSPs was 100.0%. Among all other species, the one yielding the highest score was *Pelotomaculum isophthalicicum*, which corresponded to an identity of 88.5% and a HSP coverage of 49.0%. (Note that the Greengenes databases uses the INSDC (= EMBL/NCBI/DDBJ) annotation, which is not an authoritative source for nomenclature or classification.) The highest-scoring environmental sequence was DQ378192 ('oil-polluted soil clone F28 Pitesti'), which showed an identity of 98.5% and a HSP coverage of 98.0%. The five most frequent keywords within the labels of environmental samples which yielded hits were 'microbi' (3.7%), 'anaerob' (2.9%), 'digest' (2.2%), 'soil' (2.0%) and 'thermophil' (1.7%) (213 hits in total). The five most frequent keywords within the labels of environmental samples which yielded hits of a higher score than the highest scoring species were 'microbi' (4.4%), 'anaerob' (3.3%), 'digest' (3.2%), 'soil' (2.6%) and 'condit, denitrification-induc, paddi, popul, respons, rice' (1.9%) (123 hits in total). These keywords reflect some of the ecological and physiological properties reported for strain 50-1 BON^T^ in the original description [1].

**Figure 1:**
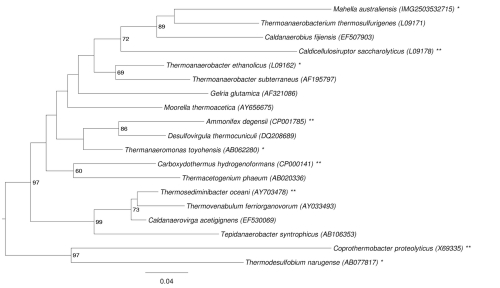
Phylogenetic tree highlighting the position of *M. australiensis* strain 50-1 BON^T^ relative to the other type strains within the order *Thermoanaerobacterales*. The tree was inferred from 1,275 aligned characters [5,6] of the 16S rRNA gene sequence under the maximum likelihood criterion [7] and rooted in accordance with the current taxonomy. The branches are scaled in terms of the expected number of substitutions per site. Numbers to the right of bifurcations are support values from 950 bootstrap replicates [8] if larger than 60%. Lineages with type strain genome sequencing projects registered in GOLD [9] are labeled with one asterisk, those registered as 'Complete and Published' with two asterisks [10,11]. Apparently, even the best BLAST hits show a low degree of similarity to *M. australiensis* (see above), in agreement with the isolated position of the species in the latest version of the 16S rRNA phylogeny from the All-Species-Living-Tree Project [12]. The species selection for Figure 1 was based on the current taxonomic classification (Table 1).

Figure 1 shows the phylogenetic neighborhood of *M. australiensis* 50-1 BON^T^ in a 16S rRNA based tree. The sequences of the three 16S rRNA gene copies in the genome differ from each other by up to two nucleotides, and differ by up to four nucleotides from the previously published 16S rRNA sequence (AY331143).

**Figure 2:**
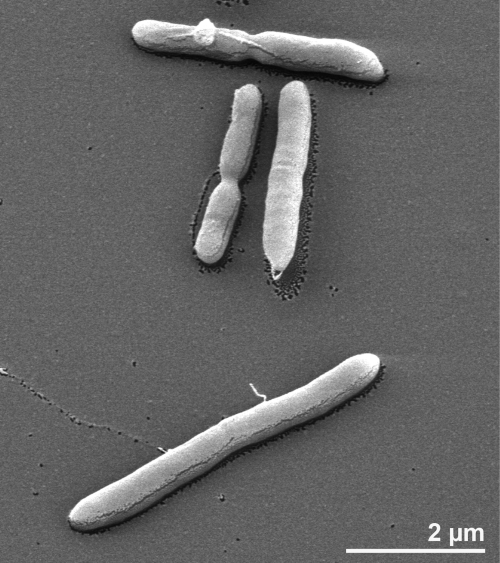
Scanning electron micrograph of *M. australiensis* 50-1 BON^T^

The cells of strain 50-1 BON^T^ are generally rod-shaped with a size of 3–20 x 0.5 µm (Figure 2). They occur singly or in pairs [1]. Strain 50-1 BON^T^ stains Gram-positive and is spore-forming (Table1). The organism is described to be motile by peritrichous flagella, with a mean of four flagella per cell [1] (not visible in Figure 2). Strain 50-1 BON^T^ was found to be a strictly anaerobic chemoorganotroph which requires 0.1% NaCl for optimal growth [1], but is also able to grow in the presence of up to 4% NaCl [1]. The organism can use a wide range of carbohydrates as carbon and energy sources, including arabinose, cellobiose, fructose, galactose, glucose, mannose, sucrose, xylose and yeast extract [1]. Lactate, formate, ethanol, acetate, H_2_, and CO_2_ are the end products of the glucose metabolism [1]. The temperature range for growth is between 30°C and 60°C, with the optimum at 50°C [1]. Mesothermophilia distinguishes *M. australiensis* from its closest relatives, such as the members if the genus *Thermoanaerobacterium* [1]. After seven days of incubation at 50°C, round colonies (1–2 mm diameter) were found in roll tubes [1]. The pH range for growth is between 5.5 and 8.8, with an optimum at pH 7.5 [1]. Strain 50-1 BON^T^ was not able to reduce thiosulfate or to hydrolyze starch [1]. Moreover, it does not use elemental sulfur, sulfate, sulfite, nitrate or nitrite as electron acceptors [1]. The generation time of the strain 50-1 BON^T^ was 11 h [1].

**Table 1:** Classification and general features of *M. australiensis* 50-1 BON^T^ according to the MIGS recommendations [13] and the NamesforLife database [14].

MIGS ID	Property	Term	Evidence code
	Current classification	Domain *Bacteria*	TAS [15]
		Phylum *Firmicutes*	TAS [16,17]
		Class *Clostridia*	TAS [18,19]
		Order *Thermoanaerobacterales*	TAS [18,20]
		Family *Thermoanaerobacteraceae*	TAS [18,21]
		Genus *Mahella*	TAS [1]
		Species *Mahella australiensis*	TAS [1]
		Type strain 50-1 BON	TAS [1]
	Gram stain	positive	TAS [1]
	Cell shape	rod-shaped	TAS [1]
	Motility	motile by peritrichous flagella	TAS [1]
	Sporulation	swollen sporangia, terminal spores	TAS [1]
	Temperature range	30°C–60°C	TAS [1]
	Optimum temperature	50°C	TAS [1]
	Salinity	0.1%-4% NaCl	TAS [1]
MIGS-22	Oxygen requirement	strictly anaerobic	TAS [1]
	Carbon source	arabinose, cellobiose, fructose, galactose, glucose, mannose, sucrose, xylose and yeast extract	TAS [1]
	Energy metabolism	chemoorganotroph	TAS [1]
MIGS-6	Habitat	oil fields	TAS [1]
MIGS-15	Biotic relationship	free-living	NAS
MIGS-14	Pathogenicity	not reported	
	Biosafety level	1	TAS [22]
	Isolation	oil well in Queensland	TAS [1]
MIGS-4	Geographic location	Riverslea Oil Field in the Bowen-Surat basin, Queensland, Australia	TAS [1]
MIGS-5	Sample collection time	1997	NAS
MIGS-4.1	Latitude	roughly -27.32	NAS
MIGS-4.2	Longitude	roughly 148.72	NAS
MIGS-4.3	Depth	not reported	
MIGS-4.4	Altitude	not reported	

Evidence codes - IDA: Inferred from Direct Assay (first time in publication); TAS: Traceable Author Statement (i.e., a direct report exists in the literature); NAS: Non-traceable Author Statement (i.e., not directly observed for the living, isolated sample, but based on a generally accepted property for the species, or anecdotal evidence). These evidence codes are from of the Gene Ontology project [23]. If the evidence code is IDA, the property was directly observed by one of the authors or an expert mentioned in the acknowledgements.

### Chemotaxonomy

No chemotaxonomic information is currently available for the strain 50-1 BON^T^.

## Genome sequencing and annotation

### Genome project history

This organism was selected for sequencing on the basis of its phylogenetic position [24], and is part of the *** G****enomic* *** E****ncyclopedia of* *** B****acteria and* *** A****rchaea * project [25]. The genome project is deposited in the Genome On Line Database [9] and the complete genome sequence is deposited in GenBank. Sequencing, finishing and annotation were performed by the DOE Joint Genome Institute (JGI). A summary of the project information is shown in Table 2.

**Table 2:** Genome sequencing project information

**MIGS ID**	**Property**	**Term**
MIGS-31	Finishing quality	Finished
MIGS-28	Libraries used	Three genomic libraries: one 454 pyrosequence standard library, one 454 PE library (10 kb insert size), one Illumina library
MIGS-29	Sequencing platforms	Illumina GAii, 454 GS FLX Titanium
MIGS-31.2	Sequencing coverage	52.1 × Illumina; 35.9 × pyrosequence
MIGS-30	Assemblers	Newbler version 2.3, Velvet, phrap
MIGS-32	Gene calling method	Prodigal 1.4, GenePRIMP
	INSDC ID	CP002360
	Genbank Date of Release	May 13, 2011
	GOLD ID	GC01760
	NCBI project ID	42243
	Database: IMG-GEBA	2503508009
MIGS-13	Source material identifier	DSM 15567
	Project relevance	Tree of Life, GEBA

### Growth conditions and DNA isolation

*M. australiensis* 50-1 BON^T^, DSM 15567, was grown anaerobically in DSMZ medium 339 (Wilkins-Chalgreen anaerobe broth, Oxoid CM 643) [26] at 50°C. DNA was isolated from 0.5-1 g of cell paste using Jetflex Genomic DNA Purification Kit (GENOMED 600100) following the standard protocol as recommended by the manufacturer. Cell lysis was enhanced by adding 20 µl proteinase K for two hours at 58°C. DNA is available through the DNA Bank Network [27].

### Genome sequencing and assembly

The genome was sequenced using a combination of Illumina and 454 sequencing platforms. All general aspects of library construction and sequencing can be found at the JGI website [28]. Pyrosequencing reads were assembled using the Newbler assembler (Roche). The initial Newbler assembly consisting of 40 contigs in one scaffold was converted into a phrap [29] assembly by making fake reads from the consensus, to collect the read pairs in the 454 paired end library. Illumina GAii sequencing data (444 Mb) was assembled with Velvet [30] and the consensus sequences were shredded into 1.5 kb overlapped fake reads and assembled together with the 454 data. The 454 draft assembly was based on 108.4 Mb 454 draft data and all of the 454 paired end data. Newbler parameters are -consed -a 50 -l 350 -g -m -ml 20. The Phred/Phrap/Consed software package [29] was used for sequence assembly and quality assessment in the subsequent finishing process. After the shotgun stage, reads were assembled with parallel phrap (High Performance Software, LLC). Possible mis-assemblies were corrected with gapResolution [28], Dupfinisher, or sequencing cloned bridging PCR fragments with subcloning [31]. Gaps between contigs were closed by editing in Consed, by PCR and by Bubble PCR primer walks (J.-F. Chang, unpublished). A total of 279 additional reactions were necessary to close gaps and to raise the quality of the finished sequence. Illumina reads were also used to correct potential base errors and increase consensus quality using a software Polisher developed at JGI [32]. The error rate of the completed genome sequence is less than 1 in 100,000. Together, the combination of the Illumina and 454 sequencing platforms provided 88.0 × coverage of the genome. The final assembly contained 364,783 pyrosequence and 4,541,603 Illumina reads.

### Genome annotation

Genes were identified using Prodigal [33] as part of the Oak Ridge National Laboratory genome annotation pipeline, followed by a round of manual curation using the JGI GenePRIMP pipeline [34]. The predicted CDSs were translated and used to search the National Center for Biotechnology Information (NCBI) non-redundant database, UniProt, TIGR-Fam, Pfam, PRIAM, KEGG, COG, and InterPro databases. Additional gene prediction analysis and functional annotation was performed within the Integrated Microbial Genomes - Expert Review (IMG-ER) platform [35].

## Genome properties

**Figure 3:**
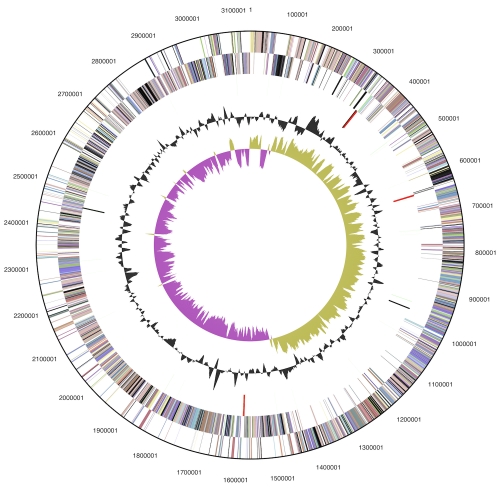
Graphical circular map of the chromosome. From outside to the center: Genes on forward strand (color by COG categories), Genes on reverse strand (color by COG categories), RNA genes (tRNAs green, rRNAs red, other RNAs black), GC content, GC skew.

The genome consists of a 3,135,972 bp long chromosome with a G+C content of 43.5% (Table 3 and Figure 3). Of the 3,033 genes predicted, 2,974 were protein-coding genes, and 59 RNAs; 104 pseudogenes were also identified. The majority of the protein-coding genes (70.4%) were assigned with a putative function while the remaining ones were annotated as hypothetical proteins. The distribution of genes into COGs functional categories is presented in Table 4.

**Table 3:** Genome Statistics

**Attribute**	**Value**	**% of Total**
Genome size (bp)	3,135,972	100.00%
DNA coding region (bp)	2,822,780	90.01%
DNA G+C content (bp)	1,362,640	43.45%
Number of replicons	1	
Extrachromosomal elements	0	
Total genes	3,033	100.00%
RNA genes	59	1.95%
rRNA operons	3	
Protein-coding genes	2,974	98.05%
Pseudo genes	104	3.43%
Genes with function prediction	2,135	70.39%
Genes in paralog clusters	103	3.40%
Genes assigned to COGs	2,154	71.02%
Genes assigned Pfam domains	2,341	77.18%
Genes with signal peptides	596	19.65%
Genes with transmembrane helices	813	26.81%
CRISPR repeats	2	

**Table 4:** Number of genes associated with the general COG functional categories

**Code**	**value**	**%age**	**Description**
J	135	5.7	Translation, ribosomal structure and biogenesis
A	0	0.0	RNA processing and modification
K	164	7.0	Transcription
L	138	5.9	Replication, recombination and repair
B	1	0.0	Chromatin structure and dynamics
D	34	1.4	Cell cycle control, cell division, chromosome partitioning
Y	0	0.0	Nuclear structure
V	59	2.5	Defense mechanisms
T	127	5.4	Signal transduction mechanisms
M	121	5.1	Cell wall/membrane/envelope biogenesis
N	57	2.4	Cell motility
Z	0	0.0	Cytoskeleton
W	0	0.0	Extracellular structures
U	51	2.8	Intracellular trafficking, secretion, and vesicular transport
O	62	2.6	Posttranslational modification, protein turnover, chaperones
C	130	5.5	Energy production and conversion
G	382	16.2	Carbohydrate transport and metabolism
E	160	6.8	Amino acid transport and metabolism
F	63	2.7	Nucleotide transport and metabolism
H	123	5.2	Coenzyme transport and metabolism
I	37	1.6	Lipid transport and metabolism
P	87	3.7	Inorganic ion transport and metabolism
Q	25	1.1	Secondary metabolites biosynthesis, transport and catabolism
R	244	10.4	General function prediction only
S	153	6.5	Function unknown
-	879	29.0	Not in COGs

## Insights from the genome sequence

### Comparative genomics

Lacking an available genome sequence of the closest relative of *M. australiensis,* (*Thermoanaerobacterium thermosulfurogenes*, Figure 1), the following comparative analyses were done with *Thermoanaerobacterium thermosaccharolyticum* (GenBank CP002171), the closest related organism with a publicly available genome. While the two genomes are similar in size (*M. australiensis* 3.1 Mb, 2,974 genes; *T. thermosaccharolyticum* 2.8 Mb, 2,757 genes), they differ significantly in their G+C content (43% vs. 34%). An estimate of the overall similarity between *M. australiensis*, *T. thermosaccharolyticum* and *Caldicellulosiruptor saccharolyticus* [11] (GenBank EKD00000000.1, as an equidistant outgroup, Figure 1), was generated with the GGDC-Genome-to-Genome Distance Calculator [36,37]. This system calculates the distances by comparing the genomes to obtain HSPs (high-scoring segment pairs) and inferring distances from the set of formulae (1, HSP length / total length; 2, identities / HSP length; 3, identities / total length). Table 5 shows the results of the pair wise comparison between the three genomes.

**Table 5:** Pairwise comparison of *M. australiensis*, *T. thermosaccharolyticum* and *C. saccharolyticus* using the GGDC-Calculator.

		HSP length / total length [%]	identities / HSP length [%]	identities / total length [%]
*M. australiensis*	*T. thermosaccharolyticum*	2.02	86.8	1.84
*M. australiensis*	*C. saccharolyticus*	1.16	86.9	1.01
*C. saccharolyticus*	*T. thermosaccharolyticum*	2.37	85.5	2.03

**Figure 4:**
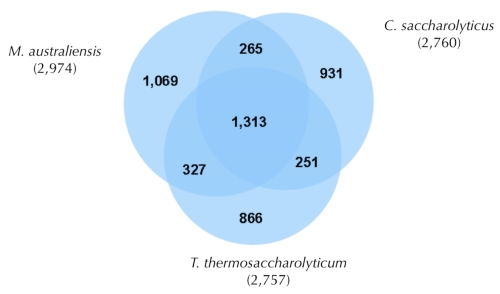
Venn diagram depicting the intersections of protein sets (total number of derived protein sequences in parentheses) of *M. australiensis*, *T. thermosaccharolyticum* and *C. saccharolyticus.*

The fraction of shared genes in the three genomes is shown in a Venn diagram (Figure 4). The numbers of pairwise shared genes were calculated with the phylogenetic profiler function of the IMG ER platform [35]. The homologous genes within the genomes were detected with a maximum E-value of 10^-5^ and a minimum identity of 30%. About half of all the genes in the genomes (1,313 genes) are shared among the three genomes, with equivalent numbers of genes (265 to 327) shared pairwise to the exclusion of the third genome or occurring in only one genome (866 to 1,069). Within the 1,069 unique genes of *M. australiensis* that have no detectable homologs in the genomes of *T. thermosaccharolyticum* and *C. saccharolyticus* (under the sequence similarity thresholds used for the comparison) the 16 genes encoding xylose isomerases appear to be noteworthy; for seven of these isomerase genes no homologs were detected in the other two genomes; only nine genes were identified in *C. saccharolyticus*, and five in *T. thermosaccharolyticum*. The high number of xylose isomerise genes suggests a strong utilization of pentoses by *M. australiensis*. It is already known that several members of the order *Thermoanaerobacterales* are capable of xylose metabolism [38]. In addition, a number of extracellular solute-binding proteins were found in the genome of *M. australiensis*. These proteins belong to a high affinity transport system, which is involved in active transport of solutes across the cytoplasmic membrane. The *M. australiensis* genome contains 54 genes coding for solute-binding proteins belonging to family 1, whereas in *C. saccharolyticus* and *T. thermosaccharolyticum* contain only 16 and 13 solute-binding protein family 1 coding genes, respectively.

*T. thermosaccharolyticum* probably transports sugars *via* a phosphotransferase system (PTS). A total of 29 genes coding for proteins belonging to the PTS specific for different sugars were found in the genome of *T. thermosaccharolyticum*. The PTS of *Thermoanaerobacter tengcongensis* was recently studied in detail [39], with 22 proteins identified as participants in the PTS. In contrast, no genes coding for PTS proteins were identified in the genome of *M. australiensis*, and only one fructose specific PEP-dependent PTS gene was reported in *C. saccharolyticus* [11]. In conclusion, the number and distribution of these transport mechanisms seems to be highly variable within the *Thermoanaerobacteraceae*.
